# Lie Symmetry Analysis and Explicit Solutions of the Time Fractional Fifth-Order KdV Equation

**DOI:** 10.1371/journal.pone.0088336

**Published:** 2014-02-11

**Authors:** Gang wei Wang, Tian zhou Xu, Tao Feng

**Affiliations:** 1 School of Mathematics and Statistics, Beijing Institute of Technology, Beijing, PR China; 2 School of Electrical Engineering, Yanshan University, Qinhuangdao, PR China; Fondazione Edmund Mach, Research and Innovation Centre, Italy

## Abstract

In this paper, using the Lie group analysis method, we study the invariance properties of the time fractional fifth-order KdV equation. A systematic research to derive Lie point symmetries to time fractional fifth-order KdV equation is performed. In the sense of point symmetry, all of the vector fields and the symmetry reductions of the fractional fifth-order KdV equation are obtained. At last, by virtue of the sub-equation method, some exact solutions to the fractional fifth-order KdV equation are provided.

## Introduction

It is well known that the Lie symmetries, originally advocated by the Norwegian mathematician Sophus Lie in the beginning of the 19th century, are widely applied to investigate nonlinear differential equations (including multi-component systems of partial differential equations (PDEs) and ordinary differential equations (ODEs)), notably, for constructing their exact and explicit solutions. Considering the tangent structural equations under one or several parameter transformation groups is the basic idea of the Lie symmetry analysis. It has been showed that how the Lie symmetry analysis have been effectively used to look for exact and explicit solutions to both ODEs and PDEs. There are a lot of papers and many excellent books (see, e.g., [1]–[20] and papers cited therein) devoted to such applications. It is important to note, however, that a very small number of them involve Lie symmetries to solve problems for fractional differential equations (FDEs).

In recent years, the investigation of FDEs has gained much attention due to an exact description of complex nonlinear phenomena in various fields: systems identification, fluid flow, control problem, signal processing, viscoelastic materials, polymers, fluid mechanics, biology, physics, engineering and other areas of science [21]–[44]. In reality, the next state of a physical phenomenon might depend on not only its current state but also on its historical states (non-local property), which can be successfully modeled by using the theory of derivatives and integrals of fractional order [38]. Given a FDEs, there exists no well-defined method to analyze and study them systematically. Also, there is no general method for dealing with exact explicit solutions to FDEs. Consequently, many powerful methods have been established and developed to construct exact, explicit and numerical solutions of nonlinear FDEs, such as Adomian decomposition method [24], [25], the invariant subspace method [26], transform method [27], [28], homotopy perturbation method [29], variational iteration method [30], sub-equation method [31]–[33], Lie symmetry group method [10]–[11], [38], [47]–[48] and so on.

In [10], the following time fractional fifth-order KdV equation
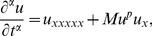
(1)was investigated by means of the Lie symmetry group method. In this paper, we aim to extend Eq. (1) to the following time fractional fifth-order KdV equation



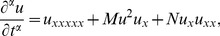
(2)where the term 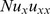 is added to (1). Here 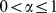, 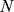 and 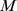 are constants. If 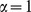, this equation can be reduced to the general fifth-order KdV equation. When 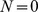, (2) can be reduced to the special case of (1). These fifth-order KdV types of equations have been derived to model many physical phenomena [20]. Recently, the authors [10] studied the time fractional generalized fifth-order KdV equation by the group classification method, the symmetries, and other properties of the equations are investigated. However, the authors did not give exact solutions of (1). Unlike the previous work, this paper will extend the work in [10] and give some exact solutions of (2). It should be noted that the above equation have several arbitrary parameters, particularly, the fractional order 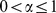, significantly affect the properties of the equation. Next, we can find that the parameters affect the symmetry and other properties of the equation, such as the symmetry reductions and so on.

Our aim in the present work is to discuss the time fractional fifth-order KdV equation by using the Lie symmetry group method. We get the corresponding infinitesimals, Lie algebra, and show that the time fractional fifth-order KdV equation can be transformed into a nonlinear ODE of fractional order. The plan of the paper is as follows. Section 2 gives some basic notions about fractional calculus and discusses the Lie symmetry analysis of the FPDEs. Then in Section 3, we perform Lie group classification on the fractional fifth-order KdV equation. In particular, some exact solutions are obtained. Finally, we present conclusions in the last section.

### Preliminaries

In this section, we give some basic notions about fractional calculus, and then we discuss the Lie symmetry analysis method to fractional partial differential equations.

### 0.1 Notations About Fractional Calculus

Here we recall definition and basic results about the recent fractional calculus, for more details we refer to [39], [40], [42]. The modified Riemann-Liouville derivative is defined by Jumarie [39]

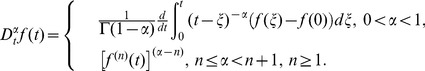
(3)


Apart from the R-L definition of fractional derivatives, there are several other definitions, for instance the modified R-L (mR-L) derivative [37], the Grünwald-Letnikov derivative (G-L) and Caputo's fractional derivative [41], [42], and so on. Under different circumstances, they can be used for handling different properties of physical models. For example, the Caputo's fractional derivative is related to initial value problems [45], [46], on the contrary, the mR-L derivative is used to investigate exact and explicit solutions of some FDEs sometimes [31]–[33].

It is simple to prove the following properties of fractional derivatives and integrals (see e.g. [39], [40]) that will be used in the analysis:
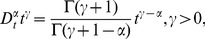
(4)

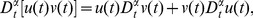
(5)

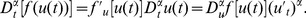
(6)


### 0.2 Lie Symmetry Analysis Method to Fractional Partial Differential Equations

We recall the main idea of this method: consider a scalar evolution equation [10], [38], [47], [48]

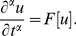
(7)


where 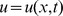 and 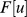 is a nonlinear differential operator.

The one-parameter Lie group of transformations
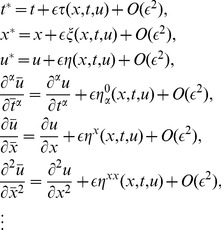
(8)where
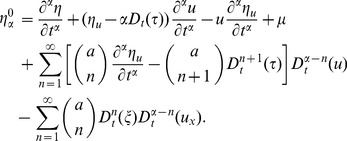
(9)here
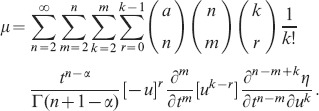
(10)

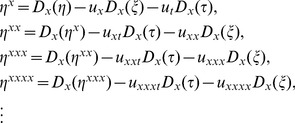
(11)


Here, 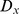 denotes the total derivative operator and is defined by
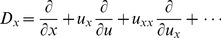
(12)


We consider the following general vector field:
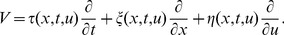
(13)


If the vector field (13) generates a symmetry of (7), then 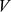 must satisfy Lie symmetry condition
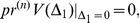
(14)


where 
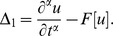


On the basis of the Lie theory, one can obtain

#### Theorem

A solution 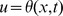 is an invariant solution of (7) if and only if

(i) 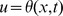 is an invariant surface, in other words,
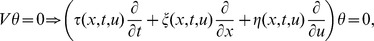



(ii) 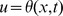 is the solution of FPDE (7).

### 0.3 Time Fractional Fifth-order KdV Equation

In this part, we determine the invariance properties of the time fractional fifth-order KdV equation. Then we construct some exact solutions of the time fractional fifth-order equation.

According to the Lie theory and the Section 2, applying the fifth prolongation 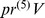 to the Eq. (2), the invariance condition (2) is equivalent to the following equation:
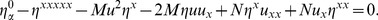
(15)


Substituting (9) and (11) into (15), and equating the coefficients of the various monomials in partial derivatives with respect to 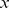 and various power of 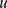, we can get the determining equations for the symmetry group of the Eq. (2). Solving these equations, one can get.
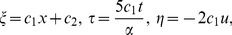
(16)


where 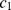 and 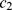 are arbitrary constants. Thus, we can get the corresponding vector fields
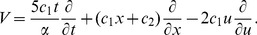
(17)


Thus, infinitesimal generators of every one parameter Lie group of point symmetries of the (2) are:
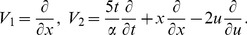
(18)


It is easily seen that the symmetry generators found in (18) form a closed Lie algebra
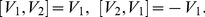
(19)


For the operator 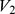 characteristic equation is
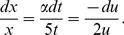
(20)


The corresponding invariants are
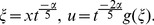
(21)


From the above discussion, one can find that (2) can be reduced to a nonlinear ODE of fractional order with a new independent variable. Consequently, we have

#### Theorem

The transformation (21) reduces (1) to the following nonlinear ordinary differential equation of fractional order
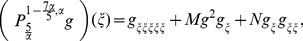
(22)


with the Erdelyi-Kober fractional differential operator 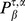 of order [44]

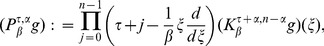
(23)

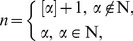
(24)where
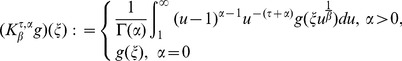
(25)is the Erd*é*lyi-Kober fractional integral operator.

#### Remark 1

The proof is similar to Theorem 2 in [10].

#### Remark 2

Although the term 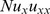 is added, the obtained point symmetries are the same as in [10].

#### Remark 3

Through the above discussion, one can find that the point symmetries of the time fractional equation are relatively narrower than those for generalized fifth-order KdV equation. The main reason is that the fractional order 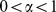 is an arbitrary parameter in our model.

### 0.4 Exact and Explicit Solutions of Time Fractional Fifth-order KdV Equation

#### 0.4.1 Summary of the method

In this part, we deal with the explicit solutions of (2) by using the improved fractional sub-equation method.

For a given NFDEs [31]–[33], [47], [48], say, in two variables 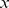 and 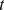,
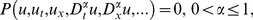
(26)


where 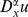 and 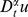 are the modified Riemann-Liouville derivatives of 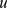 with respect to 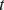 and 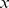, respectively.

To determine 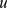 explicitly, one take the following steps:

#### Step 1

Using the variable transformation
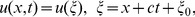
(27)


where 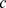 is a nonzero constant to be determined later, the fractional differential equation (FDE) (26) is reduced to a nonlinear fractional ordinary differential equation (NFODE)
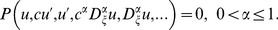
(28)


#### Step 2

Suppose that Eq. (28) has the following solution:
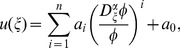
(29)


where 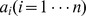 are constants to be determined later, positive integer 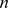 can be determined by using Eq. (26) or (28) to balance the highest order derivatives and nonlinear terms and 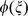 satisfies the following fractional Riccati equation:
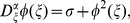
(30)


where 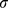 is a constant. Eq. (30) have five solutions as follows:
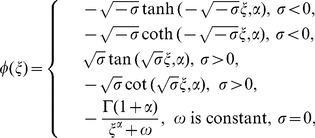
(31)


with the generalized hyperbolic and trigonometric functions
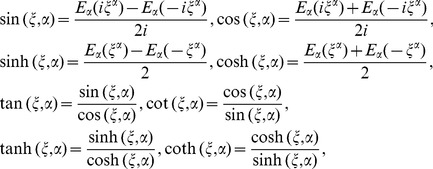
(32)


here 
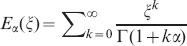
 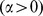 is the Mittag-Leffler function in one parameter.

#### Step 3

Substituting (31) along with (29) into (28), we can get a polynomial in 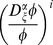. Setting the coefficients of the powers of 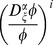 to be zero, one can obtain an over-determined nonlinear algebraic system in 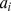 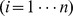 and 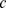.

#### Step 4

Assuming that the constants 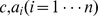 can be obtained by solving the nonlinear algebraic system in Step 3, substituting these results and the solutions of Eq. (30) into (29), one can get the explicit solutions of Eq. (26) immediately.

#### 0.4.2 Applications to Time Fractional Fifth-order KdV Equation

In this section, we apply the improved fractional sub-equation method for solving the FDEs (2).

According to above steps, we first introduce the following transformations:
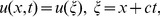
(33)


where 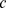 is a constant. Substituting (33) into Eq. (2), then Eq. (2) can be reduced to the following NFODE:
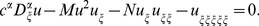
(34)


Supposing that Eq. (34) has the following solution:
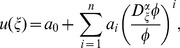
(35)


where 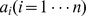 are constants to be determined later. Balancing the highest order derivative terms with nonlinear terms in Eq. (34) yields the following ansatz,
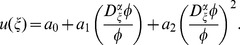
(36)


Substituting Eq. (36) along with Eq. (30) into Eq. (34) and then setting the coefficients of 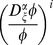 to zero, one can obtain a set of algebraic equations about 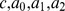. Solving the algebraic equations by Maple, we have

Case 1:
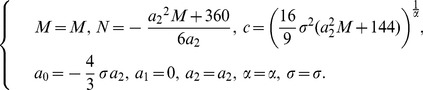
(37)


Case 2:
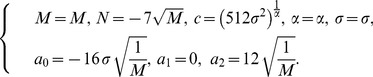
(38)


Using (37), one can get new types of explicit solutions of Eq. (2) as follows:
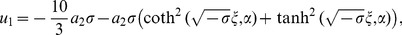
(39)


where 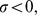 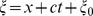.
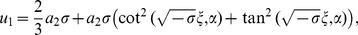
(40)


where 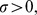 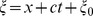.
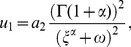
(41)


where 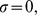 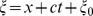.

Considering (38), one can get exact solutions of Eq. (2)
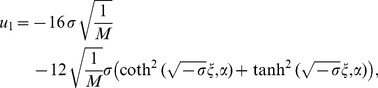
(42)


where 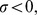 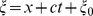.
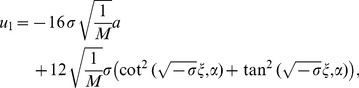
(43)


where 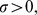 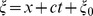.
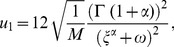
(44)


where 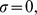 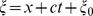.

## Conclusions

In this paper, the invariance properties of the time fractional fifth-order KdV equation are presented in the sense of point symmetry. All of the geometric vector fields and the symmetry reductions of the equation are obtained. And then, some exact solutions of the equation are constructed. The obtained solutions include generalized hyperbolic function solutions, generalized trigonometric function solutions and rational function solutions. These solutions can be further applied to deal with the nonlinear boundary-value problem, they also can be used to compare with the relevant numerical simulations. Furthermore, these solutions may be useful to further study the complicated nonlinear physical phenomena.
